# Can procalcitonin measurement help the diagnosis of osteomyelitis and septic arthritis? A prospective trial

**DOI:** 10.1186/1824-7288-35-33

**Published:** 2009-11-04

**Authors:** Sabine Faesch, Bogdan Cojocaru, Carole Hennequin, Stéphanie Pannier, Christophe Glorion, Bernard Lacour, Gérard Chéron

**Affiliations:** 1 Pediatric Emergency Department, Biochemistry Laboratory, Pediatric Orthopedic Surgery Unit, Paris-Descartes University, University of Medecine, APHP, Necker-Enfants-Malades Hospital, 149, rue de Sèvres, 75743 Paris Cedex 15, France

## Abstract

**Objectives:**

Procalcitonin (PCT) is an accurate marker for differentiating bacterial infection from non-infective causes of inflammation or viral infection. However, there is only one study in children which tested procalcitonin as a diagnostic aid in skeletal infections. With this study we sought to evaluate the sensitivity, specificity and predictive values of procalcitonin for identifying bone and joint infection in children evaluated in the emergency department for non traumatic decreased active motion of a skeletal segment.

**Methods:**

Patients aged 1 month to 14 years were prospectively included in the emergency department when suspected for osteomyelitis or septic arthritis. Procalcitonin levels, C reactiv protein, white blood cell count were measured and bacteriological samples were collected before initiation of antibiotic treatment. Patients were assigned to 3 groups according to the degree of suspected infection: group 1 confirmed infection, group 2 presumed infection and group 3 non infected patients.

**Results:**

Three hundred thirty nine patients were included (118 girls and 221 boys). Group 1 comprised 8 patients (2 had PCT levels > 0.5 ng/ml). Two had osteomyelitis and 6 septic arthritis. Forty children were incuded in group 2 (4 had PCT levels > 0.5 ng/ml). Eighteen had presumed osteomyelitis and 22 presumed septic arthritis. Group 3 comprised 291 children (9 PCT levels > 0.5 ng/ml) who recovered without antibiotic treatment. The specificity of the PCT as a marker of bacterial infection (comparing Group 1 and Group 3) was 96.9% [95% CI, 94.2-98.6], the sensitivity 25% [95% CI, 3.2-65.1], the positive predictive value (PPV) 18.2% [95% CI, 2.3-51.8] and the negative predictive value (NPV) 97.9% [95% CI, 95.5-99.2].

**Conclusion:**

PCT is not a good screening test for identifying skeletal infection in children. Larger studies are needed to evaluate still more the place of PCT measurements in the diagnosis of osteomyelitis and septic arthritis.

## Introduction

Bone and joint infections may occur at any age but they are more common in children and present a diagnostic challenge in the emergency department. Delays in diagnosis can lead to disabling sequelae. Most children diagnosed early recover completely with proper management [1].

No specific laboratory test exists for the diagnosis of bone and joint infections, with the exception of isolation of an organism from the bone or synovial fluid which is considered the gold standard, although its sensitivity ranges from 30% to 90% [2,3]. When obtained, up to 40% of blood cultures are positive, helping to identify a pathogen agent. Laboratory markers, such as elevated white blood cell count (WBC) and C-reactiv protein (CRP) levels may be helpful but are not specific [4]. Procalcitonin (PCT) serum level is very low in healthy patients (< 0.1 ng/ml) and increases rapidly in response to bacterial endotoxins [5,6]. PCT plasma concentrations are raised in severe bacterial infections (bacterial meningitis, septic shock, bacteremia and pyelonephritis), but remain fairly low in viral infections and non-specific inflammatory diseases (with a cut-off level of 0.5 ng/ml) [7-14]. In adults also, PCT is an accurate marker for bacterial infection when differentiating bacterial infection from non-infective causes of inflammation or viral infection [15-18]. Martinot, in a prospective study comparing 11 bacterial arthritis with 18 rheumatoid arthritis and 13 crystal induced arthritis, found that serum PCT is a poorly sensitive (55%) but specific (94%) marker of bacterial infection [16]. To our knowledge, there is only one study in children which tested PCT as a diagnostic aid in skeletal infections [18]. In children admitted for fever and limping, Butbul-Aviel found for PCT a sensitivity of 43.5% and a specificity of 100%. They concluded that PCT is an important informative marker in the diagnosis of osteomyelitis but not in septic arthritis. The aim of our study was to evaluate the sensitivity, specificity and predictive values of PCT for identifying bone and joint infection in children admitted in the emergency department for suspected osteomyelitis or septic arthritis.

## Materials and methods

This study was a prospective trial in which cases were collected consecutively. It was carried out between November 2004 and November 2005 in the Emergency Department of an academic tertiary care hospital in Paris, France. All the children presented in the pediatric emergency department for non traumatic decreased active motion of a skeletal segment, with or without fever (rectal temperature higher than 38°C), were prospectively studied. Children who had previously received antibiotics and newborns were excluded. All the investigators enrolling patients are emergency physicians, board certified, specialty trained in pediatrics.

The infection was suspected on clinical conditions, bacteriological findings, and laboratory markers completed by imaging study. Clinical conditions were defined as pain during passive movements, failure to move an extremity, pain, tenderness, warmth, and erythema of the involved bone, limping or decreased active motion in the involved joint, with or without fever.

Laboratory markers (PCT, WBC, and CRP) were obtained on admission, before initiation of intravenous antibiotic, when the infection was suspected. PCT was measured by an automatic quantitative method (PCT Sensitive Kryptor, BRAHMS France SAS). This assay has an improved functional assay sensitivity of 0.06 ng/ml and results ranges from 0.06 to 50 ng/ml. Values of PCT levels > 0.5 ng/ml were considered as abnormal. The results of the PCT values were obtained 24 hours after the admission, so this did not interfere with the medical decision.

Blood cultures were collected only in children with fever. Bone aspiration was performed in children suspected for osteomyelitis with a periosteal abscess on ultrasound. Joint fluid aspirations were collected in children with articular effusion, suspected on clinical and ultrasound findings, when temperature was higher than 38°C. All bacterial cultures were performed before antibiotic treatment.

A plain radiography of the affected area was performed in all patients. In all cases of children with suspected osteomyelitis, skeletal scintigraphy with 99m Tc-methylene diphosphonate was obtained. Ultrasound was performed in all children with suspected articular effusion.

Patients were assigned to three groups according to the degree of suspected infection:

### Group 1

Confirmed infection: this group consisted of patients which had one positive bacteriological culture (blood, bone aspiration or joint fluid aspiration).

### Group 2

Presumed infection: this group consisted of children with positive laboratory findings (neutrophilia > 10000/mm^3 ^and/or CRP > 20 mg/l) and either purulent bone or joint fluid aspirations or positive scintigraphy but negative cultures. Purulent aspirate was defined as cloudy macroscopic liquid and presence of deteriorated polymorphic neutrophils.

### Group 3

Non infected patients: these children had to fulfil three conditions: negative laboratory findings (all blood tests including CRP values and cultures), no X-ray or ultrasound abnormalities. Children with hip effusion, without fever and without biologic inflammatory syndrome, were classified as "transient monoarticular synovitis" and were included in group 3. All children in group 3 recovered within one week without any antibiotic treatment. Recovery was assessed one week later during an orthopedic specialist visit (blinded for PCT results).

## Results

**Figure 1:**
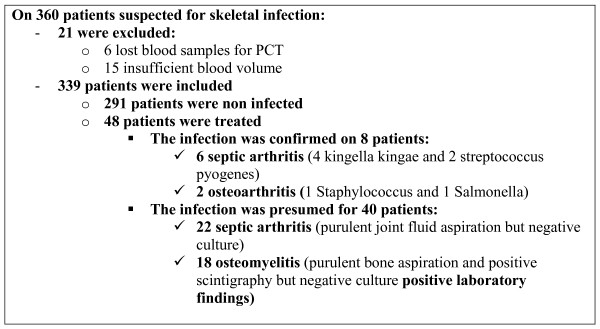
**trial profile**.

Three hundred sixty children were admitted between November 2004 and November 2005 in the pediatric emergency department for suspected osteomyelitis or septic arthritis. Twenty-one of them were excluded (lost blood samples for PCT or insufficient blood volume) [Figure 1]. Three hundred thirty nine patients, aged 1 month old to 14 years old (median 3 years, mean age 4 years), were included in the analysis (6 patients had 2 blood samples). One hundred and eighteen were girls and 221 boys.

**Figure 2:**
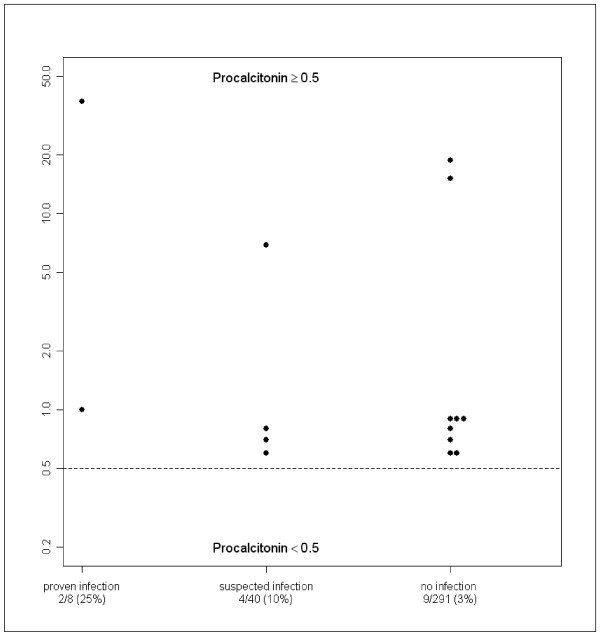
**distribution of PCT values ≥ 0.5 (in ng/ml) in the three groups**.

Group 1 comprised 8 patients with documented bacterial infection. Mean PCT level was 4.99 (± 2.3) ng/ml. Two of them (25%) had PCT levels > 0.5 ng/ml [Figure 2]. One had a multifocal osteomyelitis (PCT = 37.26 ng/ml) after varicella and one had septic arthritis of the knee (PCT = 1.01 ng/ml). The other patients had septic arthritis in 5 cases and one had osteomyelitis. There were one positive blood culture (Streptococcus), two positive bone aspirations (one Staphylococcus and one Salmonella), and five positive joint fluid aspirations (4 Kingella Kingae and 1 Streptococcus).

Group 2 comprised 40 children which were presumed infected. Mean PCT level was 0.48 (± 0.12) ng/ml. Four (10%) had PCT levels > 0.5 ng/ml (values ranges from 0.62 to 6.94 ng/ml, median 0.75 ng/ml) [Figure 2]. They all had presumed osteomyelitis with positive scintigraphy but negative bone aspiration culture. Fourteen children with presumed osteomyelitis and 22 with presumed septic arthritis had PCT levels < 0.5 ng/ml.

Group 3 comprised 291 children. Mean PCT level was 0.42 (± 0.18) ng/ml. Nine children had PCT levels > 0.5 ng/ml (values ranges from 0.59 to 18.64 ng/ml, median 0.86 ng/ml) [Figure 2]. Among them, 3 had transient acute synovitis, 3 had lower respiratory tract infection, 1 gingivitis, 1 tonsillitis and 1 was discharged with no diagnosis but recovered without antibiotic treatment.

Seventy three children had fever (6 in group 1, 23 in group 2 and 44 in group 3) and 12 of them had PCT values > 0.5 ng/ml (2 in group 1, 4 in group 2 and 6 in group 3).

**Figure 3:**
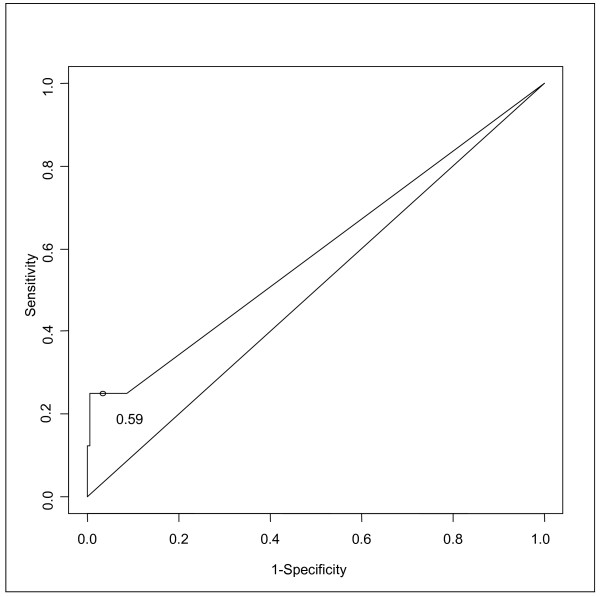
**ROC curve for PCT, proven infection versus no infection**.

**Figure 4:**
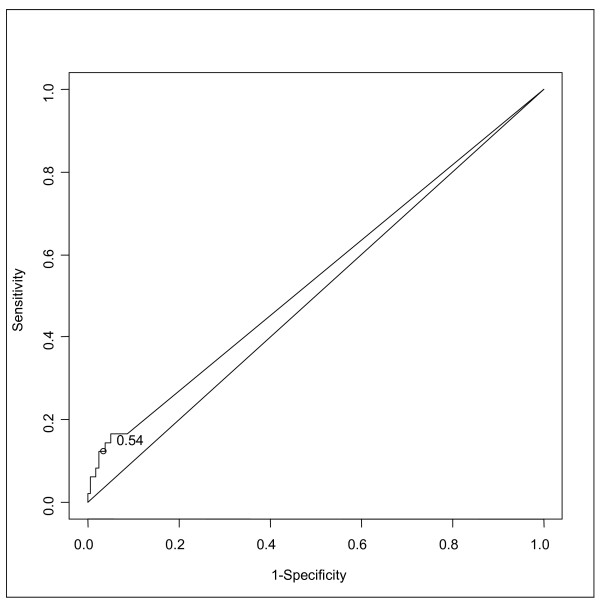
**ROC curve for PCT, proven/suspected infection versus no infection**.

In this study, the specificity (Sp) of the PCT as a marker of bacterial infection (comparing Group 1 and Group 3) was 96.9% [95% CI, 94.2-98.6], the sensitivity (Se) 25% [95% CI, 3.2-65.1], the positive predictive value (PPV) 18.2% [95% CI, 2.3-51.8] and the negative predictive value (NPV) 97.9% [95% CI, 95.5-99.2] [Table 1]. When comparing Groups 1 and 2 versus Group 3 the Sp was 96.9% [95% CI, 94.2-98.6], the Se 12.5% [95% CI, 4.7-25.2], the PPV 40% [95% CI, 16.3-67.7] and the NPV 87% [95% CI, 82.9-90.5]. When considering only children with fever, the Sp was 86.3% [95% CI, 82.3-90.2], the Se 20.7% [95% CI, 3.8-43.2], the PPV 50% [95% CI, 18.9-65.4] and the NPV 62.3% [95% CI, 31.2-82.3]. After stratification by suspected/confirmed osteomyelitis versus non-infected children, the Se was 20% [95% CI, 3.7-41.6], the Sp 96.9% [95% CI, 94.2-98.6], the PPV 30.7% [95% CI, 13.7-48.9] and the NPV 94.6% [95% CI, 91.2-97.5]. For the suspected/confirmed septic arthritis versus non-infected children, the Se was 7.1% [95% CI, 4.8-19.5], the Sp 96.9% [95% CI, 94.2-98.6], the PPV 18.1% [95% CI, 4.1-38.6] and the NPV 91.5% [95% CI, 87.4-94.3]. The receiver operating characteristic (ROC) curves comparing Group 1 versus Group 3 and Group 1 and 2 versus Group 3 are plotted in Figure 3 and 4. The area under the curves (AUC) is 0.59 [95% CI, 0.45-0.79] for proven infection versus no infection and the AUC for proven or suspected infection versus no infection is 0.54 [95% CI, 0.50-0.61].

**Table 1:** Sensitivity, Specificity, Positive Predictive Value and Negative Predictive Value of PCT with the [95% CI]

**Groups**	**Sensitivity**	**Specificity**	**PPV**	**NPV**
1 *vs *3	25 [3.2-65.1]	96.9 [94.2-98.6]	18.2 [2.3-51.8]	97.9 [95.5-99.2]

1+2 *vs *3	12.5 [4.7-25.2]	96.9 [94.2-98.6]	40 [16.3-67.7]	87 [82.9-90.5]

1+2 *vs *3 (fever)	20.7 [3.8-43.2]	86.3 [82.3-90.2]	50 [18.9-65.4]	62.3 [31.2-82.3]

1+2 *vs *3 (osteomyelitis)	20 [3.7-41.6]	96.9 [94.2-98.6]	30.7 [13.7-48.9]	94.6 [91.2-97.5]

1+2 *vs *3 (septic arthritis)	7.1 [4.8-19.5]	96.9 [94.2-98.6]	18.1 [4.138.6]	91.5 [87.4-94.3]

## Discussion

Early identification of skeletal infection is still a challenge for the clinicians, especially in the Emergency Department. Many trials found that PCT has a good specificity and a good positive predictive value for systemic bacterial infection, especially meningitis, pyelonephritis, pulmonary and neonates infections. In previously published reports, the cut off level beyond which a bacterial infection is considered as definite, ranges between 0.5 and 1 ng/ml [19,20]. In our series, with a PCT cut off level of 0.5 ng/ml, the Group 1 and 3 did not differ with a sensitivity of 25% (and only 12.5% if we consider all the infected children- Group 1 and 2 versus Group 3) and a specificity of 96.9%. Nine patients had false positive results (PCT level > 0.5 ng/ml in Group 3) and only two had positive results when infection was confirmed. Moreover, considering the ROC curves, there is no PCT cut-off level which allows to identify bone and joint infection. Our experience in the determination of the PCT levels in the initial workup of all patients admitted in the hospital with suspected bone or joint infection shows a low sensitivity (PCT level was negative in 75% of proven infection). This does not place it above clinical judgment for the correct discrimination of patients with skeletal infection. Butbul-Aviel [18] evaluated PCT values in 44 children admitted to the hospital for fever, limping and suspicion of osteomyelitis or septic arthritis. They found a higher sensitivity (58.3%) only for osteomyelitis, but the same than us for septic arthritis (27.2%). In our series, if we consider only osteomyelitis, the sensitivity is 16.6%. Their results are not confirmed by our study which may be because of our greater number of inclusions. Also we included all the patients cared for non traumatic decreased active motion of a skeletal segment, with or without fever.

Another difference with Butbul-Aviel's study is the test we used. We used a quantitative method (PCT sensitive Kryptor) characterised by a high sensitivity (0.06 ng/ml) allowing a good discrimination of values around the threshold of 0.5 ng/ml. By comparison, PCT determined by PCT- Q-test, an immunochromatographic semi-quantitative assay, is not sensitive enough to discriminate values around 0.5 ng/ml as positive or negative values.

There are several limitations to our study. The low incidence of positive bacteriological findings for the children considered as infected may be a bias. Nevertheless, bacteriologic diagnosis of orthopedic infections is known to be difficult and bacteriological samples are positive in a few numbers of cases [3,4]. On the other hand, this study was conducted in an urban teaching institution (tertiary pediatric care hospital), which has different referral patterns and serves a different population of patients than other institutions. We diagnosed 48 children as infected during this one year study, while Butbul-Aviel included 23 infected children during a two years study. Also, because there is no true gold standard for the diagnosis of skeletal infection except for positive bacteriological cultures, we applied an accepted standard supported by the literature: a combination of clinical condition, bacteriological findings, laboratory markers, completed by an imaging study when needed.

Staphylococcus aureus was described in large cohorts as the most common identifiable causative organism accounting for more than 50% of isolated organism in acute hematogenous osteomyelitis and 30% in septic arthritis [4,21]. The Staphylococcus aureus is under-represented in our case series (50% of the osteomyelitis and none of the septic arthritis) probably because of the little number (eight) of positive bacteriological findings.

Laboratory measurements should only be performed if they help making decisions about patients and/or are useful to follow the patient's evolution. Despite abundant literature on procalcitonin and its diagnostic accuracy in severe bacterial infections (bacterial meningitis, septic shock, bacteremia and pyelonephritis), in our series we found a low sensitivity (PCT level was positive in 25% of proven infection). Considering the low value of sensitivity, PCT cannot be used as a screening test for identifying skeletal infections in children. Larger studies are needed to evaluate still more the place of PCT measurements in the diagnosis of osteomyelitis and septic arthritis.

## Competing interests

The authors declare that they have no competing interests.

## Authors' contributions

SF and BC drafted the manuscript. CH and BL carried out the immunoassays. SF, BC, GC, SP and CG participated in the sequence alignment. JPJ performed the statistical analysis. SF, CG, GC conceived of the study, and participated in its design and coordination. All the authors read and approved the final manuscript.

## References

[B1] Yuan HC, Wu KG, Chen CJ, Tang RB, Hwang BT (2006). Characteristics and outcome of septic arthritis in children. J Microbiol Immunol Infect.

[B2] Timsit S, Pannier S, Glorion C, Chéron G (2005). Acute osteomyelitis and septic arthritis in children: one year experience. Arch Pédiatr.

[B3] Bonhoeffer J, Haeberle B, Schaad UB, Heininger U (2001). Diagnostic of acute haemetogenous osteomyelitis and septic arthritis: 20 years experience at the University Children's Hospital Basel. Swiss Med Wkly.

[B4] Goergens ED, McEvoy A, Watson M, Barrett IR (2005). Acute osteomyelitis and septic arthritis in children. J Paediatr Child Health.

[B5] Levine MJ, McGuire KJ, McGowan KL, Flynn JM (2003). Assessment of the test characterictics of C-reactive protein for septic arthritis in children. J Pediatr Orthop.

[B6] Assicot M, Gendrel D, Carsin H, Raymond J, Guilbaud J, Bohuon C (1993). High serum procalcitonin concentrations in patients with sepsis and infection. Lancet.

[B7] Becker KL, Nylén ES, White JC, Müller B, Snider RH (2004). Procalcitonin and the calcitonin gene family of peptides in inflammation, infection and sepsis: a journey from calcitonin back to its precursors. J Clin Endocrinol Metab.

[B8] Prat C, Domínguez J, Rodrigo C, Giménez M, Azuara M, Blanco S, Ausina V (2004). Use of quantitative and semi quantitative procalcitonin measurements to identify children with sepsis and meningitis. Eur J Clin Microbiol Infect Dis.

[B9] Pecile P, Miorin E, Romanello C, Falleti E, Valent F, Giacomuzzi F, Tenore A (2004). Procalcitonin: a marker of severity of acute pyelonephritis among children. Pediatrics.

[B10] Simon L, Gauvin F, Amre DK, Saint-Louis P, Lacroix J (2004). Serum procalcitonin and C-rective protein levels as markers of bacterial infection: a systematic review and meta-analysis. Clin Infect Dis.

[B11] Prat C, Domínguez J, Rodrigo C, Giménez M, Azuara M, Jiménez O, Galí N, Ausina V (2003). Procalcitonin, C-reactiv protein and leukocyte count in children with lower respiratory tract infection. Pediatr Infect Dis J.

[B12] Hatherill M, Tibby SM, Sykes K, Turner C, Murdoch IA (1999). Diagnostic markers of infection: comparison of procalcitonin with C reactiv protein and leucocyte count. Arch Dis Child.

[B13] Mariscalco, Michele M (2003). Is plasma procalcitonin ready for prime time in the pediatric intensive care unit?. Pediatric Crit Care Med.

[B14] Gendrel D (2004). Use of procalcitonin at the pediatric emergency. Arch Pédiatr.

[B15] Delèvaux I, André M, Colombier M, Albuisson E, Meylheuc F, Bègue RJ, Piette JC, Aumaître O (2003). Can procalcitonin measurement help differentiating between bacterial infection and other kinds of inflammatory processes?. Ann Rheum Dis.

[B16] Martinot M, Sordet C, Soubrier M, Puéchal X, Saraux A, Lioté F, Guggenbuhl P, Lègre V, Jaulhac B, Maillefert JF (2005). Diagnostic value of serum and synovial procalcitonin in acute arthritis: a prospective study of 42 patients. Clin Exp Rheumatol.

[B17] Söderquist B, Jones I, Fredlund H, Vikerfors T (1998). Bacterial or crystal-associated arthritis? Discriminating ability of serum inflammatory markers. Scand J Infect Dis.

[B18] Butbul-Aviel Y, Koren A, Halevy R, Sakran W (2005). Procalcitonin as a diagnostic aid in osteomyelitis and septic arthritis. Pediatric Emerg Care.

[B19] Lacour AG, Gervaix A, Zamora SA, Vadas L, Lombard PR, Dayer JM, Suter S (2001). Procalcitonin, IL-6, IL-8, IL-1 receptor antagonist and C-reactive protein as identificators of serious bacterial infections in children with fever without localising signs. Eur J Pediatr.

[B20] Galetto Lacour A, Zamora SA, Gervaix A (2003). Bedside Procalcitonin and C-reactive protein tests in children with fever without localising signs of infection seen in a referral center. Pediatrics.

[B21] Bonhoeffer J, Haeberle B, Schaad UB, Heininger U (2001). Diagnosis of acute haematogenous osteomyelitis and septic arthritis: 20 years experience at the University Children's Hospital Basel. Swiss Med Wkly.

